# Erratum to: *CD28/CTLA-4/ICOS* haplotypes confers susceptibility to Graves’ disease and modulates clinical phenotype of disease

**DOI:** 10.1007/s12020-017-1259-8

**Published:** 2017-02-27

**Authors:** Edyta Pawlak-Adamska, Irena Frydecka, Marek Bolanowski, Anna Tomkiewicz, Anna Jonkisz, Lidia Karabon, Anna Partyka, Oskar Nowak, Marek Szalinski, Jacek Daroszewski

**Affiliations:** 1 Department of Experimental Therapy, Hirszfeld Institute of Immunology and Experimental Therapy, Polish Academy of Sciences, R. Weigl 12, Wroclaw, 53-114 Poland; 2 Department of Endocrinology, Diabetes and Isotope Therapy, Wroclaw Medical University, L. Pasteur 4, Wroclaw, 50-367 Poland; 3 Department of Forensic Medicine, Wroclaw Medical University, M. Curie-Sklodowska 52, Wroclaw, 50-369 Poland; 4 Department of Human Evolutionary Biology, Institute of Anthropology, Adam Mickiewicz University, Umultowska 89, Poznan, 61-614 Poland; 5 Department of Ophthalmology, Wroclaw Medical University, Borowska 213, Wroclaw, 50-556 Poland

**Erratum to: Endocrine (2017) 55:186–199 DOI 10.1007/s12020-016-1096-1**

In the original publication, the copyright line in the article PDF and print is incorrectly published with Springer copyright statement instead of “The Authors”.

In addition, the license statement is missing in online and print version.

The license statement should read as:

